# Magnetic Gels in Skin Cancer Treatment: A Review of Potential Applications in Diagnostics, Drug Delivery and Hyperthermia

**DOI:** 10.3390/pharmaceutics15041244

**Published:** 2023-04-14

**Authors:** Marcos Luciano Bruschi, Glécilla Colombelli de Souza Nunes

**Affiliations:** Laboratory of Research and Development of Drug Delivery Systems, Postgraduate Program in Pharmaceutical Sciences, Department of Pharmacy, State University of Maringa, Av. Colombo 5790, Maringa 87020-900, PR, Brazil; profglecillacolombelli@gmail.com

**Keywords:** nanomedicine, drug delivery systems, magnetic gels, hydrogels, magnetic nanoparticles, theranostic, targeted therapy, chemotherapy, hypothermia

## Abstract

Skin cancer (SC) is affecting an increasing number of people worldwide. Its lesions affect mainly the most exposed regions of the skin. SC is classified into to main categories: non-melanoma (basal cell carcinoma of the epidermis and squamous cell carcinoma) and melanoma (the abnormal proliferation of melanocytes, which is rarer, more hazardous, and more deadly). Prevention and early diagnosis are important actions, and surgery is often considered. After the removal of cancerous lesions, the local administration of medicine can guarantee anticancer therapeutic action, rapid healing and the recovery of tissue, ensuring the absence of recurrence. Magnetic gels (MGs) have attracted increased attention regarding their pharmaceutical and biomedical applications. They are magnetic nanoparticles (e.g., iron oxide nanoparticles) dispersed in a polymeric matrix, which constitute adaptive systems under a magnetic field. MGs can combine magnetic susceptibility, high elasticity, and softness, and are thus useful platforms for diagnostics, drug delivery, and also for hyperthermia. This manuscript reviews MGs as a technological strategy for the treatment of SC. An overview of SC and the treatment, types, and methods of preparing MGs are discussed. Moreover, the applications of MGs in SC and their future perspectives are considered. The combination of polymeric gels and magnetic nanoparticles continues to be investigated, and new products must hit the market. Clinical trials and new products are expected, due to the important advantages of MGs.

## 1. Introduction

The natural aging of human beings can lead to many disorders. One of the most notorious signs that time has elapsed is related to changes that are reflected in the appearance of the skin and mucous membranes, characterized by alterations in their structures and functions [1,2,3]. The skin is one of the largest organs of the human body and is responsible for many functions; these include receiving information through sensory nerve endings, and contributing to the thermoregulation of the body and the excretion of substances through its blood vessels, glands, and adipose tissue. This organ has the important function of regulating water loss and protecting the body against micro-invaders, carcinogens, and mechanical aggressions; in addition, it produces and accumulates melanin, with a protective function against ultraviolet rays [4,5,6,7,8].

The action of physical, chemical, and biological agents can lead to serious problems, such as injuries, alterations, and the destruction of the skin. Mechanical and chemical aggressions, the presence of microbial infections, and the appearance of malignant cellular alterations and transformations (e.g., cancer) are factors that can lead to the morbidity of the skin [2,7].

Cancer is the second leading cause of mortality after cardiovascular diseases, and is the main cause of death in over 50 countries. In 2020, there were nearly 10 million deaths from cancer globally, which is expected to increase to 16 million by 2040 [8]. Skin cancer is one of the most common and serious types [9,10,11]. Among the different types of cancer, it is a worldwide health problem that has significant adverse effects on the population’s health. As such, the number of people affected by this problem increases rapidly compared with other types of cancer [9,10,12]. Cancerous lesions affect mainly the most exposed regions of the skin, such as the back, shoulder, forearm, face, and ears.

Skin cancer’s classification is based on two groups, melanoma and non-melanoma, the first of which is used for cases that are more rare, hazardous, and deadly [7,10,13]. Melanoma is the 17th most common cancer worldwide, the 13th most common in men, and the 15th most common in women. Australia and New Zealand are the two countries with the highest rates of both types of skin cancer [14].

Prevention and early diagnosis are important actions to avoid the progression of this type of cancer [5,7]. Moreover, several advances have been made through traditional treatment methods, such as chemotherapy, immunotherapy, radiotherapy, photodynamic therapy (PDT), and surgery [13,15]. However, the prospects for healing are not always good. Additionally, multiple adverse effects and complications have been reported. In the worst cases, local disfigurement can occur, such as on the face, motor functions such as speech, chewing, and swallowing can be lost, and even in milder cases, mucositis and candidiasis can occur [13,15,16].

Surgery is often considered the standard protocol for the treatment of certain types of skin cancer, such as non-melanoma, because it guarantees the histopathological control of the lesion. After the removal of cancerous lesions, the local administration of medicine that guarantees anticancer therapeutic action is also extremely important; this is associated with the rapid healing and recovery of the affected region. It is crucial that the complete recovery of the tissue is ensured, especially for elderly patients who experience slower repair. In addition, it is very important to ensure the absence of recurrence, thus avoiding a new wound resection [5,13].

Therefore, the development of pharmaceutical formulations aimed at administering biologically active agents to the skin for protecting (preventing), maintaining, and treating these problems is of huge importance [5]. The suitable selection of both the pharmaceutical dosage form, considering the route of administration, and the biologically active agent, is an essential requirement for effective therapy [17,18,19].

Many biologically active agents of natural or synthetic origin and technologies have been proposed for the prevention and adjuvant therapy of the different types of skin cancer. They have displayed important therapeutic results because of their antioxidant, antimicrobial, cytotoxic (directly or using PDT), protective and recovery properties and their effects on injured tissues [5,13,15,20].

The strategies and technologies aiming to modify the drug release have evolved quickly, enabling the development of specialized therapeutic systems [21,22,23]. In particular, the use of nanostructured materials, obtained from the dispersion of different phases, and with properties that respond to environmental conditions, can increase the therapeutic efficacy and patient adherence to the therapy [18,19,24,25].

In recent decades, magnetic nanostructured systems have attracted increased attention regarding their pharmaceutical and biomedical applications, such as in biosensors, theragnostics, drug delivery, and in antimicrobial and antitumoral functions [25,26,27]. In particular, gel materials that can exploit shape transitions when submitted to a magnetic field have been proposed for the treatment of cancer [27,28]. Magnetic gels or ferrogels are adaptive systems under a magnetic field. They can undergo coiling, torsion, elongation, rotation, or bending, which disappear quickly as the magnetic stimulus is removed. These transitions can also be controlled by remote access, allowing contact with the deepest tissues of the body in order to perform the temporal and spatial control of drug delivery. Magnetic gels can combine magnetic susceptibility, high elasticity, and softness, constituting useful platforms for diagnostics, drug delivery, and also for treatments using hyperthermia [28,29].

Therefore, it is very important to explore the strategy used to apply magnetic gels to skin cancer treatment, with an overview of the up-to-date and most employed technologies for skin cancer prevention as either main or auxiliary therapy. This manuscript provides a comprehensive review of the utilization of magnetic gels as a technological strategy for skin cancer treatment. Conceptual information about magnetic gels, their main compositions, physicochemical characteristics, properties useful for application in skin cancer treatment, their advantages and disadvantages, the state-of-the-art, and their market is addressed.

## 2. Skin Cancer

Cancer was described by Hippocrates as the formation of a tumor showing the capacity to resemble crabs due to its projections, and used the Greek word for crab *carcinos* to describe it. Later, the Latin word “cancer” started to be used with the same meaning [30]. There are several types of cancer involving different tissues and organs of the human body, and skin cancer is one of the most common [9].

The skin can receive information from nervous sensory endings, enabling thermoregulation and substance excretion through its blood vessels, glands, and adipose tissue. The skin’s main function is the protection of the organism against environmental agents and also the production of melanin for protection against ultraviolet rays (UV) [2]. The skin’s structure is composed of the stratum corneum, epidermis, dermis, and hypodermis. The skin’s stratum corneum and epidermis constitute the most external region, which is non-vascularized and is formed by epithelial tissue, comprising keratinocytes, melanocytes, Langerhans cells (intraepidermal macrophages), and Merkel cells (tactile epithelial). The epidermis is about 20 cells thick, is composed of four to five sublayers of keratinocytes (stratified squamous epithelial cells), and can provide protection against the external environment. This structure ranges from 75 to 150 µm in thickness, reaching up to 0.6 mm on the palms of the hands and soles of the feet. The epidermis is also composed of cells that are responsible for the pigmentation of the skin (melanocytes) and the protection of the immune system [4]. All this structure is maintained using oxygen and nutrients supplied by capillary loops from the dermis. Moreover, tactile sensations (e.g., touch, pressure, pain, and temperature) are provided to the epidermis by nerve receptors. There is a sophisticated interlocking organization between the epidermis and dermis, and this region is called the dermal–epidermal junction. The dermis, or corium, shows a great amount of extracellular matrix, constituted by conjunctive tissue, with high amounts of collagen, proteoglycans, fibronectin, and elastin. Moreover, appendages such as sweat glands, hair follicles, sebaceous glands, and nerves, are in the dermis (reticular region) [4]. The hypodermis, also called subcutaneous or adipose tissue, is the deepest layer. It is composed of connective and adipose tissues, lymphatic and blood capillaries, and nerve endings [2].

The integrity of this system provides protection to the body against irritants, pathogens, and water loss. Moreover, the body’s thermoregulation is maintained by sweating and vasodilatation, shivering, and vasoconstriction [2]. However, age and environmental factors (chemical, biological and physical agents) can change this integumentary system and lead to an infectious process, the destruction of tissues, and other deleterious processes in the skin [1,2]. Aging causes structural and functional skin changes. For example, xerosis cutis, pressure ulcers, and skin tears are common among elderly people. Microorganisms, sun damage, and noxious agents can stress the skin and lead to carcinogenesis [1].

Skin cancer is one of the most prevalent cancers and is considered a multistage process that involves three steps: initiation, promotion, and progression [3]. After exposure to a carcinogenic factor, damage to cellular deoxyribonucleic acid (DNA) can occur. For example, UV radiation can induce the carcinogenic process when photons damage DNA and affect membranes, proteins, and DNA via reactive oxidative stress. The unrepaired DNA damage results in permanent irreversible genetic mutations, which induce autonomous cellular growth. After this process of initiation, the promotion step starts when these changed cells are continuously submitted to substances that promote the benign process (tumor) of selective clonal cell proliferation. This process may be promoted by the presence of UV radiation, wounding, chronic inflammation, and oxidative stress associated with the regeneration process. During the progression stage, the tumor can undergo more genetic mutations and experience an increase in its invasive capacity. The result is a malignant neoplasm that shows metastasizing ability. The preexisting vascular structures can create new blood vessels to maintain the nutrition and oxygen supply to the tumor, enabling its exponential growth in the progression stage [5,9,13].

Skin cancer is the most common type of cancer and has an increasing incidence, mainly in regions with a higher number of white populations. It may be classified as nonmelanomatous or melanomatous. The nonmelanomatous type is more prevalent and originates from epithelial (keratinized) cells, and it may be divided into squamous cell carcinoma (SCC) and basal cell carcinoma (BCC) [13]. Laënnec (1806) was the first researcher to describe melanoma as cancer caused by a malignancy of melanocytes. Further, the BCC and SCC cancer types were described by Jacob Bowen in 1827 and 1912, respectively [30].

Together, BCC and SCC represent 95% of the occurrence of skin cancers. The most common form of any skin cancer is the BCC, displaying slow-growing and locally invasive characteristics [13]. BCC is more frequent than SCC and grows from the epidermis (basal cells), being located mainly in the face and head. The second most common form of nonmelanomatous skin cancer is the SCC, which is responsible for about 20% to 30% of cases [13]. SCC is generated by irregularly proliferated malignant cells in the epidermis. These cells can metastasize to lymph nodes or other tissues across the body. There is an SCC subtype that is considered to be precancerous called actinic keratosis. It appears as an erythematous papule and may be caused by the same risk factors previously discussed, such as chemicals and UV light [20]. In general, the risk of developing a type of nonmelanomatous skin cancer is related to a variety of factors; however, exposure to ultraviolet (UV) radiation is considered the main one [16].

Melanomataous skin cancer (or melanoma) is a therapy-resistant malignant tumor of melanocytes that accounts for about 2% of skin cancer; however, it causes the most deaths. Melanoma is more common among white people and its incidence is quickly increasing worldwide, resulting in public health problems [13]. Possessing fair skin and experiencing a chronic exposure to sunlight constitute the main risk factors. There is a direct relationship between UV light exposure and the development of melanoma. Skin protection from UV light is essential, and most of the time, this type of skin cancer requires treatment from pathologists, surgeons, and cancer specialists [31]. The incidence of melanoma is higher in males than in females, and it is increasing in children. Furthermore, the back in males and the arms and legs in females are the most common areas in which tumors present. The face is a common region as well, and early detection leads to the increased survival of patients. Tumors that are localized only to the epidermis do not show a risk of death and present a low risk of metastasis [31,32]. Therefore, early detection is fundamental and can minimize metastatic disease; overall, prevention is fundamental [32,33]. It is essential to avoid intense and intermittent sun exposure, thus reducing contact with UV light. Some actions, such as the self-examination of skin and the investigation of risk factors, are essential as well. The early diagnosis and treatment of melanoma are also fundamental. The development of effective treatment can lengthen the survival time of patients displaying the advanced disease [7,32,33].

Melanomatous or nonmelanomatous tumors are serious issues and many early detection techniques for skin cancer have been developed. Moreover, it is fundamental to determine the lesion characteristics, such as shape, color, symmetry, size, and location. This information is fundamental to detecting the disease and distinguishing benign skin cancer from melanoma [7].

## 3. Treatments of Skin Cancer

The best procedure for treating an initial melanoma tumor is surgical excision; however, chemotherapy and adjuvant therapy, considering the use of vaccines, when possible, are additional treatments for melanomas in advanced stages [31,32,33]. Radiotherapy and chemotherapy are the main and traditional strategies for the treatment of cancer. However, skin cancer is less responsive to these methods, which result in unsatisfactory therapies without lasting effects [20]. Moreover, these methods are unable to target the cancerous cells and the normal ones are destroyed as well.

Considering the characteristics of SCC, BC, and melanoma tumors, skin cancer constitutes a worldwide problem, and new technologies for the prevention and therapy, both adjunct and main, of skin cancer, are necessary. Nowadays, the treatment of skin cancer involves a variety of approaches and technological strategies. They are composed of surgical procedures (excision with standardized margin or Mohs micrographic surgery), radiation, chemotherapy (topical and systemic), electrodessication and curettage, cryotherapy, and photodynamic therapy. The choice of therapy is dependent on the cancer type, the patient’s suitability and preference, the expertise of the health professional, and the availability of local services [15,34].

The surgical procedure remains the main choice for the treatment of any type of skin cancer. Both the excision, Moh’s micrographic surgery, and curettage and electrodessication need adjunct or complementary therapies in most cases, such as the topical administration of medicines for wound healing, pain relief, and protection [34].

Chemotherapy and radiation therapy are also common and traditional treatments, mainly when surgical procedures are not possible, in the case of low-risk tumors, and when the patient cannot undergo surgery. The administration of drugs that are able to destroy the cancer cells or the application of radiation in order to lethally the target cancer cells via DNA damage constitute important strategies. The cell exposition to radiation can activate many complex signaling cascades, which can result in cell cycle arrest or cellular apoptosis. This radiation exposure can produce the activation of various DNA repair mechanisms, in order to avoid the downstream effects on untreated neighboring cells [15,34].

Synthetic or biologically active compounds from plants have been shown to be beneficial against skin cancer because they can exert anti-carcinogenic action. These anticancer properties are due to their anti-oxidative, anti-proliferative, anti-inflammatory, and anti-angiogenic effects [5]. The topical administration route is the ideal route of drug delivery in skin cancer treatment. However, the chemoprotective efficacy of these compounds in skin tumors when administered via the oral route has been supported by studies and evidence as well. In this case, their protective effects are very dependent on pharmacokinetics and their bioavailability in the body [5].

For the topical administration of both phytochemicals and synthetic drugs, it is necessary to consider several issues, such as the skin penetration capacity, the formulation stability, the bioactive agent concentration, and also the duration of the treatment. Moreover, controlled drug delivery through the topical or oral route, and also the interaction of bioactive compounds with alternative therapies, should be investigated [5].

The localized delivery of cytotoxic compounds constitutes a great benefit, and can avoid or limit systemic toxicity and enhance patient compliance in the treatment. Specifically, having to bypass through the *Stratum corneum* and epidermal barriers is a limiting factor for topical administration. Moreover, the necessity of targeting the cancer cells, avoiding the death of normal cells, and also developing more effective and safer therapies more, justifies the investigation and development of new strategies and technologies. In this context, magnetic gels have been proposed for the treatment of skin cancer [34,35].

## 4. Magnetic Gels

### 4.1. Properties of Magnetic Materials

Magnetic materials are widely used for the development of new technologies. In the environmental area, for example, these materials have been exploited to remove heavy metals and organic matter from water [36]; in the area of biotechnology, iron oxides have been used as heat delivery agents in magnetic hyperthermia systems, and also for the vectorization of drugs in drug delivery systems [26,34,37,38,39,40].

The behavior of magnetic materials depends on the response of atomic magnetic dipoles when subjected to the application of an external magnetic field. These behaviors can be classified as follows: diamagnetic, paramagnetic, ferromagnetic, antiferromagnetic, and ferrimagnetic [41,42,43].

The magnetic phenomenon known as diamagnetism exists in all materials, but it is so weak that it normally cannot be observed when the material has one of the other properties, such as paramagnetism or ferromagnetism [41]. The magnetic susceptibility (χ), which is a quantitative measure of the magnetization that the material presents as a function of the applied field, of a diamagnetic material is negative [41,42].

Paramagnetic materials are characterized by a positive and small magnetic susceptibility, and a linear response that is proportional to the applied field [41,42]. These materials do not present resultant magnetic moments, since the magnetic moments of the atoms do not interact with each other and also do not show directional preferential alignment; as a consequence, zero resultant magnetization is observed. However, when a magnetic field is applied to paramagnetic materials, there is a tendency for the magnetic moments to align with the applied magnetic field [41,42].

**Figure 1:**
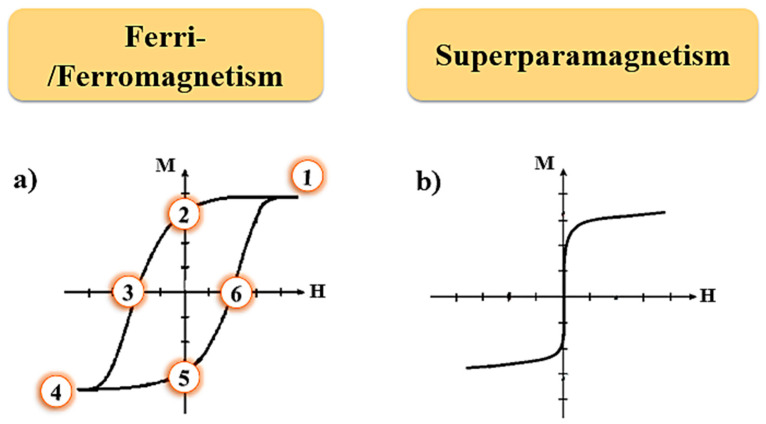
Magnetization curves versus applied magnetic field (M vs. H): (**a**) Typical magnetic hysteresis that occurs in both ferromagnetic and ferrimagnetic materials, where positions 1 and 4 represent the saturation magnetization, and positions 2 and 5 represent the remanence of material; (3) and (6) correspond to the coercivity in two opposite directions of the applied magnetic field; (**b**) Behavior of a superparamagnetic material.

On the other hand, ferromagnetic materials can maintain a resulting magnetization in the absence of an applied external field, because their dipoles tend to orient themselves in the same direction as the field, and when this occurs, the magnetization of the material increases up to a saturation limit [41,42,43]. Once this limit is reached, if the external field is removed, the magnetization returns to the remaining magnetization point. These materials are characterized by presenting a spontaneous magnetization below a certain temperature, which is known as the Curie temperature; below this temperature, the materials are classified as ferromagnetic and above this temperature, as paramagnetic. Ferromagnetic materials have χ ≫ 1 [41,42]. Figure 1a displays the typical behavior of ferromagnetic materials, in which a hysteretic behavior is observed.

Ferrimagnetic materials present adjacent magnetic moments in opposition and at different intensities, resulting in a net magnetic moment, which has its origin in the incomplete cancellation of the spin moments. Both ferromagnetic and ferrimagnetic materials, below the Curie temperature, are composed of regions called magnetic domains and exhibit magnetic hysteresis (Figure 1a) [41,42]. In these regions, there is a mutual alignment of all magnetic dipoles in the same direction. Each domain is separated from one other using boundary walls, also called domain walls. Therefore, ferromagnetic and ferrimagnetic materials present magnetic hysteresis curves when subjected to an external magnetic field due to the movement of these boundary walls [41,42].

Finally, antiferromagnetic materials are those that present the phenomenon of the pairing of magnetic moments between adjacent atoms or ions. Thus, the spin magnetic moments of neighboring atoms or ions are in opposite directions. An example of an antiferromagnetic material is manganese oxide (MnO). The magnetic susceptibility of these materials is greater than one [41,42].

The magnetic properties of a material can be modified by changing its dimensions from a macroscopic to a nanometric scale. Materials whose dimensions are on the nanometric scale are called nanomaterials. Nanomaterials are composed of nanoparticles (NPs) [44].

In particular, nanoparticles of ferromagnetic or ferrimagnetic materials exhibit a magnetic behavior called superparamagnetism (the exact size limit at which superparamagnetism occurs depends on the material composition, particle size, and temperature) [45,46]. A superparamagnetic particle is a single-domain system that has its magnetization reversed spontaneously. In superparamagnetism, particles have an intense and rapid collective response to external magnetic fields. While they have an intense response to external fields, they do not have a collective magnetic moment when the external field is turned off. Therefore, as they do not remain magnetized after the external magnetic field ceases, the magnetic nanoparticles (MNPs) do not interact with each other, forming clusters of particles [41,45,46].

Figure 1 shows the typical magnetization curve of superparamagnetic NPs. As the magnetic field increases, the magnetic domains align until the moment of magnetic saturation is reached. When decreasing the value of the magnetic field, the magnetization decreases, not showing coercivity.

The action of a magnetic field can cause MNPs to produce heat (magnetic hyperthermia), and magnetic hyperthermia has been used to treat cancer [38,47,48,49], as tumor cells are more sensitive to heat than healthy cells [38]. Duan and coworkers [50] discussed the influence of the electromagnetic field on the development of melanoma, and concluded that the electromagnetic field not only influenced the development of melanoma, but also had a remarkable impact on the absorption of anticancer drugs and clinical therapies for melanoma.

Recently, the progress of MNPs for the treatment of cancer has mainly involved remote control through a magnetic field, either alternating or static [28,51]. Static magnetic fields can, for example, direct MNPs to the desired location, offering an attractive combination of on-demand and site-specific drug delivery. The alternating magnetic field can incorporate the NPMs into a thermosensitive polymeric matrix; for example, converting the external magnetism into local heat induces the release of drugs [52].

Some MNPs have already been approved by the United States’ regulatory agency the Food and Drug Administration (FDA), and are marketed and used in medicine. For example, Feraheme (ferumoxytol), which is an MNP product that makes it possible to treat some types of anemia linked to chronic kidney disease, was approved in 2009 by the FDA [39,53].

The most researched MNPs for the area of nanomedicine are based on iron oxides, such as magnetite (Fe_3_O_4_) and maghemite (γ-Fe_2_O_3_), as they are biodegradable when exposed to biological systems, have low toxicity and good biocompatibility with the body [28,39]. However, the MNP of iron oxides is not stable in the blood or in aqueous environments, because alone they are prone to oxidation due to their large surface area and chemical activity, which compromise their magnetism and dispersibility [39].

One of the ways to get around the problem of oxidation of iron oxide MNPs is to disperse (or encapsulate) them in a polymeric matrix or in gels [26,44]. To encapsulate the MNPs, a polymer that is capable of “masking” the physicochemical characteristics of the encapsulated particles is chosen in order to increase their circulation time in the blood and promote their chemical interaction with the cells [54].

According to Allen and coworkers [18], a gel is a semi-solid pharmaceutical form of one or more active principles, composed of appropriate gelling agents whose purpose is to give firmness to a solution or colloidal dispersion. A gel must have at least two components, a network and a solvent. The network is a series of polymer chains connected at crosslinking points to form a 3D structure [55,56]. The threads of the polymeric network can surround themselves with solvent molecules, pushing away the neighboring chains so that the network occupies a larger volume and so that the solvent causes the network to swell [28,56,57]. This swollen network is what is called a gel [57].

Therefore, magnetic gels are structured heterogeneous systems that contain magnetic particles dispersed in three-dimensional networks of polymeric chains [56,58].

Some of the techniques used to characterize magnetic gels include carrying out analyses to determine their magnetic properties, e.g., using a vibrating sample magnetometer (VSM); scanning electron microscopic analyses (SEM), used to analyze particle morphology; transmission electron microscopy analyses (TEM), used to determine particle size (a very important factor in achieving superparamagnetism); X-ray diffraction analysis (XRD), used to verify the crystalline structure of MNP and impurities in the material; rheological analyses (rotational and extensional), used to determine the behavior of magnetic gels under a certain shear rate; and an analysis of viscosity, the viscoelasticity modulus (G and G’) and material behavior through Young’s modulus (stress–strain curves [57,58]).

### 4.2. Preparation Methods of Magnetic Gels

Three methods are distinguished in the literature for the preparation of magnetic gels: (i) the direct blending method; (ii) the in situ method; and (iii) the grafting-onto method [27,44,59,60,61]. The method for preparing the gel is based on the properties of the MNPs and the polymeric network of the gel, as well as on the homogeneity of the concentration and distribution of the NPs in the gel [27,28,61].

In the direct blending method, the gel and the MNPs are prepared separately and then mixed [27,44,61]. For example, one can prepare Fe_3_O_4_ NPs via the coprecipitation method [44], and then the prepared Fe_3_O_4_ NPs must be dispersed in an aqueous or oily solution (so-called ferrofluid mixture) to avoid the oxidation and aggregation of the Fe_3_O_4_ NPs. Finally, the mixture of ferrofluid and the gel precursor solution are crosslinked, resulting in the encapsulation of the NPMs [28,43]. In this method, the crosslinking of MNPs with the gel can be performed by adjusting the concentration of the reagents, or even the speed of stirring [27,44,61]. The difficulty of this method is to achieve a uniform distribution of NPMs within the gels, as these can interfere with the formation of the network and the final structure of the gel [27,43]. In addition, NPMs can diffuse out of magnetic gels when immersed in a liquid solution, and NPMs do not interact with the gel network [27].

Mikhnevich and coworkers [62] developed poly (acrylamide) ferrogels using a direct blending method. The authors used the free radical polymerization of the acrylamide monomer and N, N’-methylene-bis (acrylamide) as a crosslinking agent in the presence of magnetic nickel nanoparticles. Shi and coworkers [63] also employed this method to prepare a mixture of bisphosphonate (BP)-modified hyaluronic acid polymeric solution and iron oxide (Fe_3_O_4_) nanoparticle dispersion, in which the hydrogel networks are crosslinked by BP groups and iron atoms on the surface of the particle.

In the in situ method, the synthesis of MNPs is carried out within the gel network [27,44,61]. The advantage of carrying out the preparation of MNPs together with the gel is the control that is afforded over the nucleation and growth of the magnetic particles due to the constraints of the polymeric network [28]. In this method, the gels are first manufactured; then, the gels are placed in a concentrated aqueous solution containing Fe^+2^ or Fe^+3^, and the ferrous ions are “taken up” in a 1:2 molar ratio until equilibrium is reached [43,64,65]. Finally, the gel, which is swollen due to the absorption of ferrous ions, is immersed in an alkaline solution so that the NPs precipitate [61]. Therefore, this method is limited to hydrogels that have stable networks, as they can be destroyed in the alkaline solution [43].

Sang and coworkers [66] developed magnetic gels based on poly(2-acrylamido-2-methyl-1-propanesulfonic acid) (PAMPS) and iron oxide nanoparticles using this method. The researchers immersed the hydrogel in a concentrated aqueous solution of ferric and ferrous ions, followed by precipitation with an aqueous ammonia solution. Singh and coworkers [67] used this method to synthesize magnetic iron oxide hydrogel nanocomposites that were incorporated into chitosan–graphene oxide, aiming to investigate the efficiency of this method for the preparation of the aforementioned nanocomposites to be used as an adsorbent for the removal of a cationic dye.

In the grafting-onto method, covalent bonds are formed between the gel network and the NPMs, grafting several functional groups at the surface of the MNPs; these function as nano-crosslinkers to form a covalent coupling with the monomers when polymerized [27,44,61]. Among the mentioned methods, this one has a complex manufacturing process, a long preparation cycle, and a high cost, which all limit its applications.

**Figure 2:**
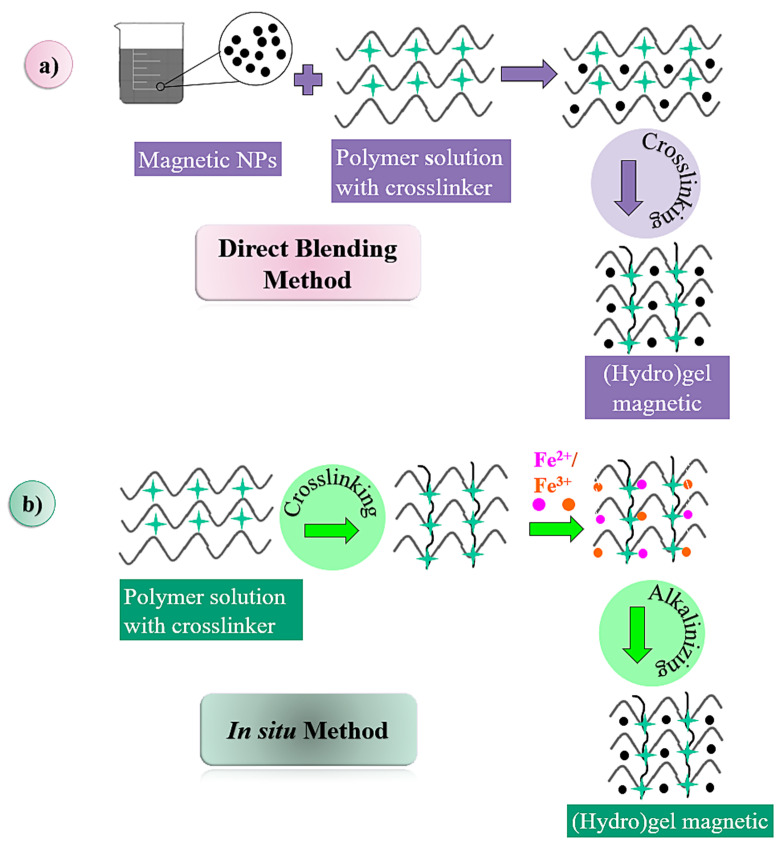

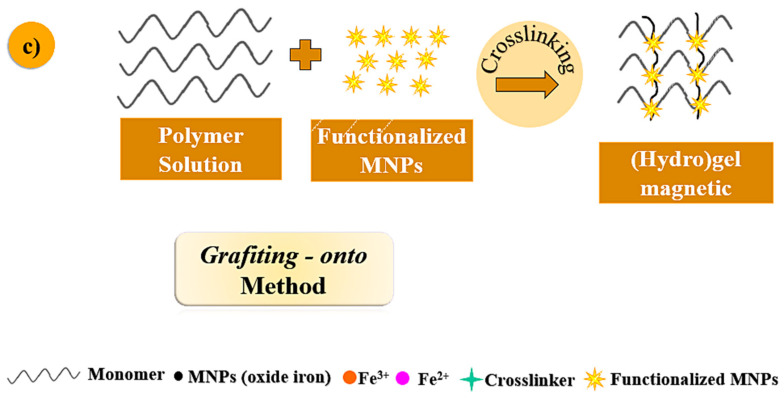
Schematic representation of the methods used to prepare magnetic gels: (**a**) direct blending; (**b**) in situ; (**c**) grafting-onto method.

Figure 2 shows a schematic representation of the methods used to prepare magnetic gels.

## 5. Magnetic Gels for Skin Cancer

The treatment of skin cancer is dependent on various factors and is based on surgical excision, radiotherapy, and chemotherapy [13,15,31,35,57]. These are the common and traditional procedures; however, this type of cancer can show a strong resistance and less sensitivity to chemotherapeutic drugs and radiation, leading to unsatisfactory results and effects that are not prolonged [20,31]. Another problem is the low selectivity of these therapies, showing weak specificity toward the diseased cells and destroying the normal ones [20].

Biochemotherapy using interleukin 2, or using both interferon alpha and interleukin 2 together with some chemotherapy regimens, have shown to be successful strategies, and may display a complete response after long-term treatment [31]. Moreover, phytochemicals have shown to be beneficial for the treatment of skin cancer, acting as anti-carcinogenic agents. They act due to anti-oxidative, anti-inflammatory, anti-angiogenic and anti-proliferative biological effects. Moreover, they are widely available, cost-effective and highly tolerated [5].

Photodynamic therapy (PDT) is another promissory strategy for the therapy of skin cancer tumors, displaying better long-term therapeutic capabilities compared to those previously described. PDT can be used for a level of better selectivity, avoiding the destruction of normal intact cells. It is based upon the ability of photosensitizers to be activated by a predetermined light wavelength in order to produce singlet oxygen and/or reactive oxygen species; this is so that a phototoxic effect that leads to the oxidation of the signaling pathways in the cellular environment or to the regulation of gene expression is established. This therapy constitutes an important strategy and may be used together with others, such as nanotechnology [20].

Nanotechnology has shown to be of great importance in cancer treatment, enabling the development of materials and systems for improved therapies with greater selectivity and safety for the patients. Nanostructured systems have displayed the ability to pass through biological barriers and improve the bioavailability of drugs in the targeted tumor cells. Moreover, low drug doses, a reduction in the side effects, and an increase in the treatment efficiency are observed [20,35]. Nanoparticles have been utilized as theragnostic systems that display targeted and improved drug delivery in skin cancer therapies. Their advantages can be summarized as follows: they improve selectivity and drug delivery to the tumor cells, improve the effect of enhanced permeability and retention (EPR effect), reduce adverse effects, improve the aqueous solubility of drugs, and can increase the half-life of anticancer drugs [20,34]. Lipid-based nanoparticles (solid lipid nanoparticles and nanostructured lipid carriers), liposomes, polymeric nanoparticles, micelles, dendrimers and inorganic or metallic nanoparticles (gold nanoparticles and silica nanoparticles) constitute the main types that have been studied for the therapy of skin cancer [68]. Despite the low number of studies on the safety of some of these systems, they have shown to be fundamental to the dermal delivery of anticancer drugs, gene and immune therapies. The use of vaccines for skin cancer (especially for malignant melanoma) has partially reduced the incidence of tumors and has increased the immune response of patients. This enables active and specific immunotherapy, and endows the host with tumor antigens [35].

**Figure 3:**
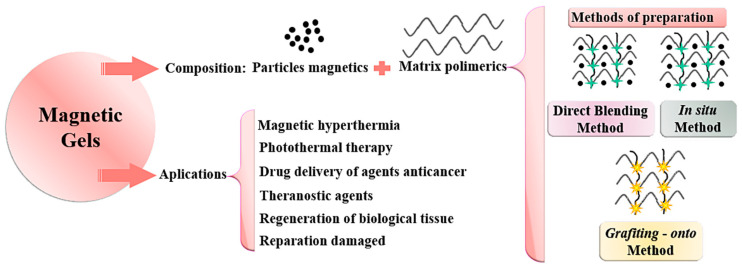
Schematic representation of the applications of magnetic gels and methods of preparation.

Currently, new forms of treatments are being investigated, either alone or applied in combination with those mentioned above, that aim to aid the healing process when the diseased tissue is removed [27,61,63,69]. Among the new strategies proposed is the use of magnetic gels that aim to alleviate the side effects of treatments (Figure 3), providing specificity directed at the injured tissue, and greater effectiveness and safety [69]. However, one of the main difficulties encountered when using these systems is inability of MNPs to penetrate due to the barrier function of the skin, although the barrier is partially broken in lesions or during inflammatory episodes, as in the case of skin cancer [34].

Thus, in order to present this topic and give an overview of the recent developments in the formulation of the magnetic gels used in skin cancer treatment, bibliographical research was carried out in the following databases: PubMed, Scopus, Web of Science, Science Direct, BioMed Central (BMC), Google Scholar, and Scielo. The following keywords and Boolean operators were used: skin cancer AND magnetic gel, skin cancer AND hydrogel, skin cancer AND melanoma, and skin cancer AND magnetic hydrogel. Selected results were based on their titles, abstracts, and full-text availability. Priority was given to publications from the last ten years.

Most studies show work in the development phase and present in vitro and in vivo (animals) characterizations that have not yet been clinically tested. An example of research on the development of magnetic gels is the study of Daya and coworkers [39], who synthesized MNPs with curcumin (CMNP) using the method of coprecipitation. The authors dispersed CMNPs in hyaluronic acid (HyA) to create angiogenic magnetic hydrogels targeting tissue repair. The magnetic hydrogels developed in this work were shown to have desirable cytocompatibility and angiogenesis properties with magnetic guidance, which is promising for improving tissue regeneration. The research conducted by Daya and coworkers [39] promises a new form of treatment that can be used, for example, in combination with those already mentioned in order to aid healing when the diseased tissue is removed during the treatment of skin cancer.

In addition, we found that several works are seeking the development of magnetic gels in order to treat other types of cancer. For example, Xie and coworkers [69] developed injectable magnetic gels using commercial chemicals for application in the magnetic hyperthermia of tumors in vivo. The researchers added commercial magnetic metals (metal oxide powders) to a Ca^2+^-alginate (ALG-Ca^2+)^ gel. In vitro tests were performed with 4T1-type cells, and in vivo tests were performed using Kunming rats. According to the researchers, the gel facilitated the accumulation of metal powders at the tumor site after the peritumoral injection, significantly improving the precision and efficiency of the treatment and reducing the risk of toxicity to the surrounding tissues. In addition, it was verified in the work that the magnetic gel had an excellent therapeutic effect and thus could be used for application in magnetic hyperthermia. Another example is the study by Jahanban-Esfahlan and coworkers [70], which aimed to design and develop a pH-responsive natural magnetic hydrogel based on alginate (Alg), gelatin (Gel), and Fe_3_O_4_ magnetic nanoparticles as a delivery system (DDS) for the treatment of cancer with chemotherapy. Based on the results, the magnetic hydrogel, Alg-Gel/Fe_3_O_4_, developed in work can be considered an efficient drug delivery system for cancer therapy and diagnosis. Furthermore, in vitro tests were performed using HeLa cells; the researchers found that the cytotoxicity of the Alg-Gel/Fe_3_O_4_ magnetic hydrogel loaded with Dox showed excellent potential as a DDS for cancer chemotherapyand as a magnetic property for diagnosis via MRI technique.

Thus, this study has verified that the development of magnetic gels directed at the treatment of skin cancer is still in the stage of searching for the best magnetic nanocompounds and in that of performing cell tests (in vitro, in vivo and ex vivo) in order to verify the biocompatibility, cytotoxicity, and efficacy of these materials. However, no information was found directly describing clinical studies focusing on the use of magnetic gels for the treatment of skin cancer. Table 1 summarizes other studies on the development of magnetic nanocomposites and performance studies (in vitro, in vivo, and ex vivo) conducted in order to ascertain their effectiveness against cancer cells.

## 6. Future Perspectives

Skin cancer is a class of diseases that has a high incidence and can result in severe complications in humans that can lead to death. New pharmaceutic dosage forms must be developed and reach the market in order to improve the efficiency, safety, and quality of skin cancer treatment.

Smart drug delivery systems such as magnetic gels can improve the diagnosis, prevention, and therapy of skin cancer. Magnetic gels can contribute to controlled targeted drug delivery, improving wound healing, control, and the treatment of tumors and skin infections, for example, using photodynamic therapy and hyperthermia. Moreover, the possibility of their activation via the application of a remote magnetic field corroborates the applicability of their use.

Many proposed magnetic gels are composed of biocompatible and biodegradable polymers, showing a high potential for pharmaceutical and biomedical applications. Magnetic gels can display very important properties for skin cancer treatment, such as a rapid response to magnetic fields, porosity, extracellular matrix mimicking, low toxicity, and biocompatibility, constituting an important advance in technological strategy for pharmaceutical and biomedical application.

In the last decade, many studies have shown the potential application of magnetic gels in the treatment of skin cancer, as an important strategy and technological innovation. In hyperthermia therapy, magnetic gels may constitute an efficient strategy for improved radio/chemo due to its improved targeted drug delivery, protecting the health of skin cells. Many formulations of magnetic gels are being developed as multi-component systems, constituting products for utilization in different types and stages of skin cancer.

However, the performance of magnetic gels is dependent on their composition, physicochemical composition, magnetic response, cytotoxicity, and safety. Some of these aspects are not clear yet. The place and future of magnetic nanoparticles in the skin are not understood. Studies of pharmacokinetics, metabolism, and in vivo biodegradation are necessary for a better understanding of these systems [27,55]. These points may restrict the application of magnetic hydrogels in the clinic.

## 7. Conclusions

Studies of and developments in new magnetic gel products for skin cancer are growing in number and quality. However, the application of magnetic gels in clinics is dependent on these challenges previously reported. The existence of few devices and the low number of studies involving the development of magneto-induction devices for human tests may constitute another issue impairing the utilization of magnetic gels in clinics. In the next few years, clinical trials and the development of market formulations of magnetic gels for skin cancer treatment are expected to take place at a higher velocity. It is very hard to identify studies of these products in humans and in different stages/phases. Therefore, the combination of polymeric gels and magnetic nanoparticles continues to be investigated, and new products must hit the market. Clinical trials and new products are expected, due to the important advantages of these systems.

## Figures and Tables

**Table 1:** Summary of other studies that developed magnetic nanocomposites using iron oxides, test types and cell lines.

Materials	Cell Lines	Type of Test	Reference(s)
Fe_3_O_4_ NPs (20–30 nm) and PEG hydrogel (80PEG1000MA, 50PEG1000MA, and 20PEG1000MA gels were fabricated with 80, 50, and 20 mol% PEG1000MA macromer to PEG400DMA crosslinker).^1^ And the solution of Taxol^®^ were utilized in the hydrogels for cell tests	Glioblastoma (M059K), breast adenocarcinoma (MDA MB 231), and lung carcinoma (A549) cells	in vitro	[49]
Nanoclusters composed of iron oxide Fe_3_O_4_ NPs (mean size of about 8 nm) crystallographically aligned	Tumors extracted from mice challenged with B16-F0 cells (murine melanoma, ATCC CRL-6322)	in vitro ex vivo in vivo	[71]
Hybrid nanogels designed by coating bifunctional nanoparticles with a thermo-responsive poly(N-isopropylacrylamide-co-acrylamide) [poly(NIPAM-AAm)]-based hydrogel as the shell plus curcumin	Melanoma cells B16F10	in vitro	[72]

^1^ PEG (N = 1000) methyl ether methacrylate (PEG1000MMA) and crosslinker PEG (N = 400) dimethacrylate (PEG400DMA).

## Data Availability

The data that support the findings of this study are available from the corresponding author upon reasonable request.
